# An Integrated, Multimodal, Digital Health Solution for Chronic Obstructive Pulmonary Disease: Prospective Observational Pilot Study

**DOI:** 10.2196/34758

**Published:** 2022-03-17

**Authors:** Brian D Gelbman, Carol R Reed

**Affiliations:** 1 Division of Pulmonary and Critical Care Medicine Weill Cornell Medical Center New York, NY United States; 2 Wellinks (Convexity Scientific, Inc) New Haven, CT United States

**Keywords:** COPD, patient engagement, mHealth, digital health, mobile phone, telemedicine, mobile apps, remote monitoring, spirometry, pulse oximetry

## Abstract

**Background:**

Chronic obstructive pulmonary disease (COPD) affects millions of Americans and has a high economic impact partially due to frequent emergency room visits and hospitalizations. Advances in digital health have made it possible to collect data remotely from multiple devices to assist in managing chronic diseases such as COPD.

**Objective:**

In this pilot study, we evaluated the ability of patients with COPD to use the Wellinks mHealth platform to collect information from multiple modalities important to the management of COPD. We also assessed patient satisfaction and engagement with the platform.

**Methods:**

A single-site, observational, prospective pilot study (N=19) was conducted using the Wellinks platform in adults with COPD. All patients were aged over 30 years at screening, owned an iPhone, and were currently undergoing a treatment regimen that included nebulized therapy. Enrolled patients received a study kit consisting of the Flyp nebulizer, Smart One spirometer, the Nonin pulse oximeter, plus the Wellinks mHealth app, and training for all devices. For 8 weeks, participants were to enter daily symptoms and medication use manually; spirometry, nebulizer, and pulse oximeter data were automatically recorded. Data were sent to the attending physician in a monthly report. Patient satisfaction was measured via a 5-point scale and the Net Promoter Score (NPS) captured in interviews at the end of the observation period.

**Results:**

Average age of the patients was 79.6 (range 65-95) years. Participants (10 female; 9 male) had an average FEV_1_% (forced expiratory volume in 1 second as % of predicted for the patient) of 56.2% of predicted (range 23%-113%) and FEV_1_/forced vital capacity of 65%. COPD severity, as assessed by the Global Initiative for Chronic Obstructive Lung Disease (GOLD) classification, was mild in 2 patients, moderate in 6, and severe/very severe in 11; 9 patients were on home oxygen. During this 8-week study, average use of the spirometer was 2.5 times/week, and the pulse oximeter 4.2 times/week. Medication use was manually documented 9.0 times/week, nebulizer use 1.9 times/week, and symptoms recorded 1.2 times/week on average. The correlation coefficients of home to office measurements for peak flow and FEV_1_ were high (r=0.94 and 0.96, respectively). Patients found the app valuable (13/16, 81%) and easy to use (15/16, 94%). The NPS was 59.

**Conclusions:**

This study demonstrates that our cohort of patients with COPD engaged with the Wellinks mHealth platform avidly and consistently over the 8-week period, and that patient satisfaction was high, as indicated by the satisfaction survey and the NPS of 59. In this small, selected sample, patients were both willing to use the technology and capable of doing so successfully regardless of disease severity, age, or gender. The Wellinks mHealth platform was considered useful and valuable by patients, and can assist clinicians in improved, timely decision making for better COPD management.

## Introduction

Chronic obstructive pulmonary disease (COPD) is a respiratory disorder characterized by persistent symptoms such as shortness of breath, coughing, excess mucus production, and irreversible expiratory airflow obstruction. It primarily affects people aged 65 and over and is often a result of exposure to risk factors such as tobacco smoke or air pollutants [1]. In 2018 in the United States, 16.4 million people reported having a diagnosis of COPD, chronic bronchitis, or emphysema, according to the Centers for Disease Control and Prevention’s National Health Interview Survey [2] and COPD was the fourth leading cause of death in the United States [3].

COPD has a high financial impact. COPD-related costs in the United States were projected to be US $49.0 billion in 2020, including direct health care expenditures and indirect morbidity and mortality costs [4]. COPD exacerbations, during which symptoms of coughing and shortness of breath acutely worsen, are a significant driver of these expenditures, due to emergency room visits, hospital admissions, relapses, and readmissions [5]. COPD has a 30-day readmission rate of approximately 20% and is a target condition in the Medicare Hospital Readmission Reduction Program [6]. Frequent COPD exacerbations can lead to a decline in lung function and quality of life and may significantly affect a patient’s prognosis [7,8].

The current standard of care for COPD includes medications, smoking cessation, oxygen supplementation (when indicated), and pulmonary rehabilitation [9]. At each office visit, patient biometric data are collected (eg, pulse oximetry and spirometry) and the COPD care plan is communicated. Between office visits, however, it is difficult for the physician to gather insights into patient behavior and adherence to the care plan; significant clinical deterioration or noncompliance can go undetected. Furthermore, many patients with COPD have limited mobility due to the debilitating nature of their lung disease, making office visits challenging or not possible at all. Currently there are few comprehensive and reliable tracking solutions to assess patient adherence to the care plan or the impact of medication and other interventions on a more frequent basis [8,10,11].

Advances in digital health have now made it possible to share medical information in real time, thus enabling more immediate management of chronic diseases. Remote patient monitoring solutions have been successfully developed in other chronic diseases, such as congestive heart failure and diabetes [12,13]. During the COVID-19 epidemic, patients of all demographic groups quickly adopted some form of technology to access medical care [14,15]. Monitoring modalities are now being evaluated for factors such as ease of integration into existing clinician workstreams, correlation with traditional diagnostic measures and treatment modalities, and overall patient acceptance [16].

For patients with COPD, effective remote monitoring should include information on medication adherence and symptoms, plus biometric data including spirometry and pulse oxygen [17]. However, the ability to monitor data streams from different digital sources has presented some challenges, such as accuracy, measuring adherence, and ease of use for both the patient and the clinician, including incorporation into existing electronic medical records [8,11,17].

In this pilot study, we evaluated the Wellinks mHealth platform in a small cohort of patients with COPD. Our objective was to, within a trial setting, determine the feasibility of the platform to track information important to the management of COPD, collected via Bluetooth from multiple modalities including spirometry and pulse oximetry, medication adherence, and patient input of symptoms and medication use. This small-scale study was performed to obtain feedback on patient satisfaction and engagement with the platform, and to inform modifications needed for larger clinical studies.

## Methods

### Study Design

This prospective observational pilot study of the Wellinks mHealth Platform was conducted from January to May 2021 at a single outpatient pulmonary practice. A sample size of 20 patients was planned. Monitoring data were collected over an 8-week-per-study-patient observation period. Informed consent was obtained from each participant, and the study was conducted according to the principles stated in the Declaration of Helsinki (2013). Patients were deidentified of protected health information according to the Safe Harbor method of the Health Insurance Portability and Accountability Act (HIPAA). Each patient was assigned a unique study ID number and the principal investigator maintained sole access to reidentification codes.

### Ethical Approval

This study was approved by the Institutional Review Board (IRB) (IRB#:20-WELL-101) with respect to the scientific content and ethical treatment of human research participants.

### Recruitment

Participants were recruited by the principal investigator within the clinical setting. Inclusion criteria specified male or female patients with COPD over 30 years of age with English language literacy who were prescribed a treatment regimen that included nebulized therapy. All patients had access to an iPhone running iOS version 13.4 or later.

Exclusion criteria were acute or chronic conditions that might interfere with patients’ ability to participate in the study, or comorbidities that might interfere with data collection and interpretation (eg, renal, cardiac, hepatic, central nervous system, or psychiatric conditions).

Participation in this study was completely voluntary. Participants were permitted to keep the items in the study kit. No additional financial inducements were offered, and no patient recruitment materials were used.

### Patient Use of the Intervention

**Figure 1:**
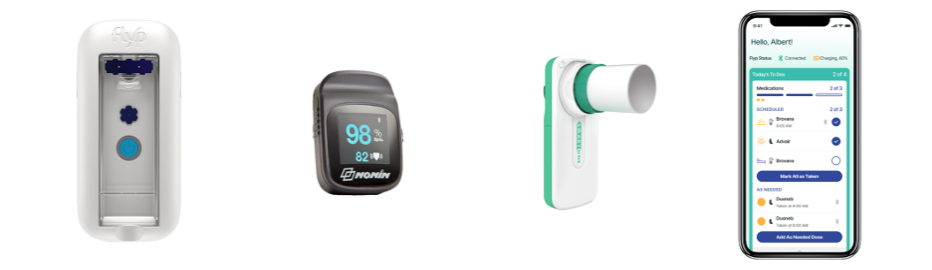
Components of the Wellinks mHealth Kit, including the Flyp nebulizer, MIR Smart One spirometer, and Nonin pulse oximeter, are pictured here from left to right. Patients used their own phones to download the Wellinks mHealth App.

After informed consent was obtained, participants received the study kit, which consisted of 3 devices: the Flyp nebulizer, MIR Smart One spirometer, and Nonin pulse oximeter, plus instructions for use (Figure 1). In addition, participants received instructions for downloading the Wellinks mHealth iOS app. The physician or another member of the research team explained the function of all devices and the app. Participants were considered enrolled once the app was downloaded and the participant had logged in.

Patients were asked to perform forced expiratory volume in 1 second (FEV_1_) and pulse oximetry measurements (peripheral oxygen saturation or SpO_2_) at least once weekly; these data were automatically recorded in the app via Bluetooth. Patients’ use of the Flyp nebulizer was also automatically recorded; most patients were prescribed nebulizer treatments as rescue therapy only.

**Figure 2:**
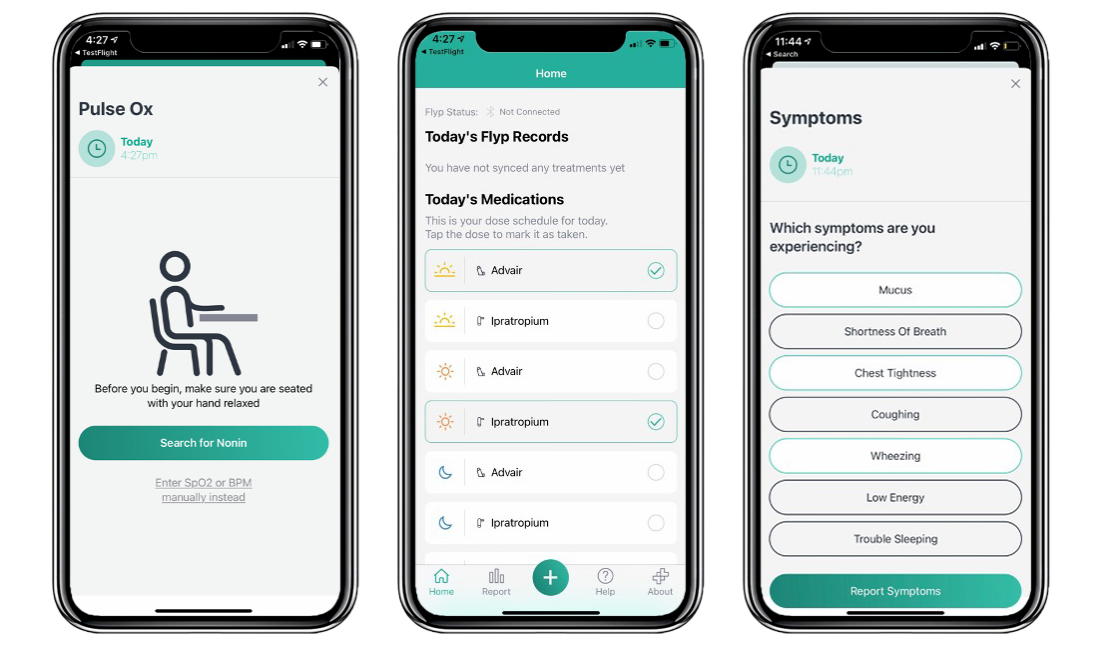
The app displays content that guides the patient while recording pulse oximetry, or inputting medications taken and symptoms experienced from a list. The medication list was prepopulated with each patient's prescribed medications.

Daily medication usage and symptoms were entered into the app manually by the patient (Figure 2). The app was prepopulated with a customized list of medications prescribed to each patient for COPD, including dosing intervals; patients were able to check off when the dose of each prescribed medication was taken. Patients could also enter the use of additional medications, such as rescue inhalers or nebulizer treatments. A list of symptoms (mucus production, shortness of breath, chest tightness, wheezing, coughing, low energy, and trouble sleeping) allowed patients to either check the symptom boxes or to check a “no symptoms” box if they were not having symptoms. Patients were not prompted by the app or the clinical research team to use the devices or to enter information into the app.

**Figure 3:**
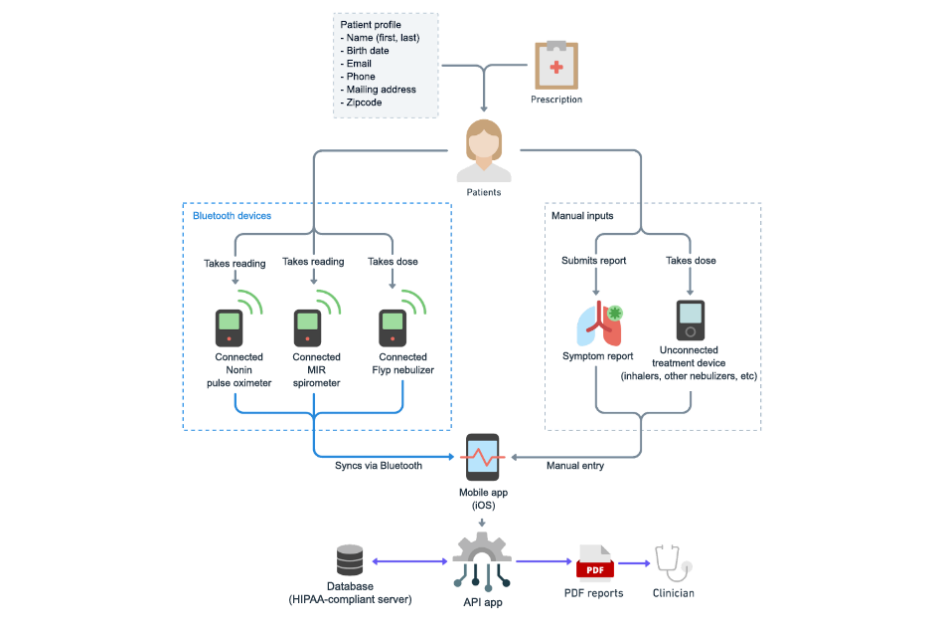
The Wellinks mHealth platform recorded spirometry, pulse oximetry, and nebulizer use automatically by bluetooth. The patient manually input medications taken and symptoms experienced (if any) using the app. Data were stored securely and compiled into a summary Portable Document Format (PDF) report for the clinician monthly. API: application programming interface, HIPAA: Health Insurance Portability and Accountability Act.

The Wellinks app collects and stores data related to treatment and respiratory status of the patient on a secure, HIPAA-compliant, cloud-hosted database. Monthly reports containing a summary of the collected medication adherence data, FEV_1_ and SpO_2_ measurements, and symptoms or notes were sent to the clinician via secure email. Any subsequent visits or treatment were to be consistent with current standard of care (Figure 3).

### Patient Engagement and Satisfaction

Patient engagement was evaluated by assessing how often patients used the various features of the Wellinks mHealth platform over the 8-week study period. For spirometry and pulse oximetry, the baseline case was 1 use/week. Use of the medication and symptoms recording feature was left to the patient’s discretion.

At the end of the observation period, patients’ overall attitudes about and perception of the Wellinks mHealth solution were assessed using a 9-question 5-point Likert scale (responses ranged from 1=strongly disagree to 5=strongly agree). In addition, patients had the opportunity to provide qualitative feedback to the interviewer regarding their experience and the intervention.

The Net Promoter Score (NPS) was used to gauge patient satisfaction overall with the Wellinks mHealth solution. The NPS is derived from the answer to just 1 question: “On a scale of 0-10, how likely is it that you would recommend using the Wellinks app to friends, family members, or associates also living with COPD?”. The score is determined by subtracting the percentage of “Detractors” (those who gave a score of ≤6) from the “Promoters” (those who gave a score of 9 or 10). The “Passives” (those who gave a score of 7 or 8) are thought to be satisfied with the product, but not to the point of recommending the product or “promoting” it to others [18,19]. With the methodology used here, the total score (tallied from all responses) will fall between –100 and 100, with –100 being the worst outcome and 100 being the best. This survey was administered to the patient by video call or telephone by a member of the Wellinks research team.

**Figure 4:**
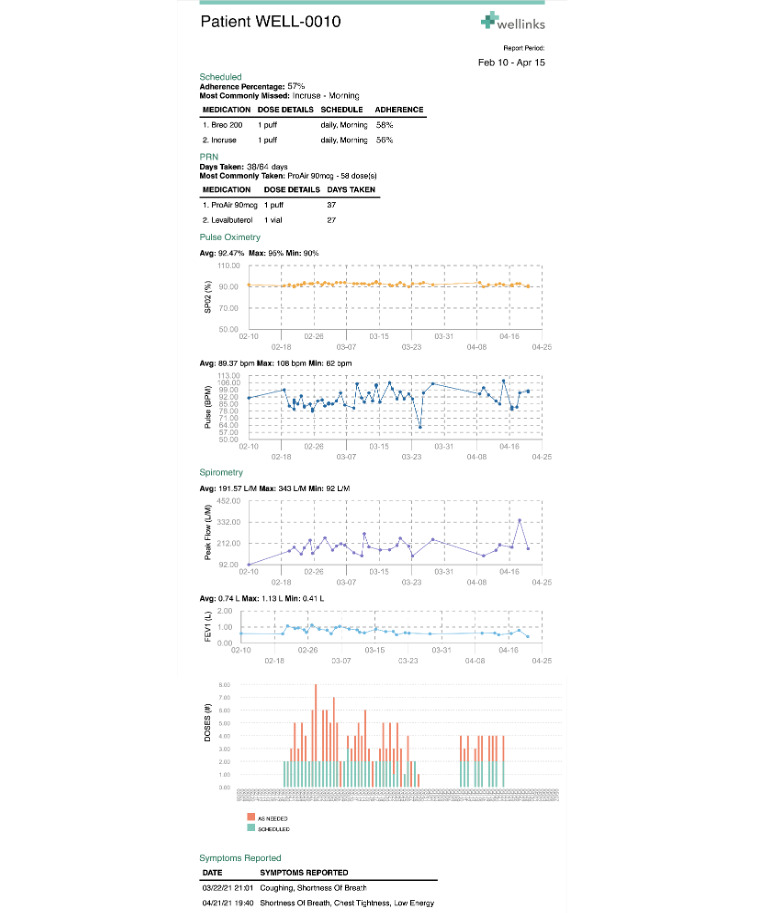
The physician report supplied monthly summarizes prescribed scheduled medications, PRN medications and adherence, pulse oximetry and spirometry readings over time, and symptoms. PRN: pro re nata.

The Wellinks mHealth platform generated a physician report monthly (Figure 4). Each report was customized for each patient, and provided the following summary of that patient’s activity in the preceding month:

- Scheduled medications (name and dosage): adherence percentage and medication most commonly missed;
- As-needed (pro re nata [PRN]) medications (name and dosage): how often and how much taken;
- SpO_2_% over time;
- Pulse (beats/minute) over time;
- Spirometry (peak flow and FEV_1_) over time; and
- Symptoms: list of symptoms experienced and when entered (date and time).

### Adverse Events

Adverse events occurring during the study period were documented by the investigator; these were not reported to the device manufacturer unless they were considered serious and related to the device. Serious adverse events were to be reported using an unanticipated device effects form and were to be reported to the sponsor and to the IRB.

### Analysis

Data were summarized with means and ranges for quantitative variables with normal distribution (eg, age, spirometry values). The Pearson correlation coefficients were calculated to compare concordance between home and office spirometry; mean values from the Smart One spirometer were compared with FEV_1_ values collected at the closest patient visit date (office spirometry was performed on a Vyaire Vmax Encore). Correlations between office and home pulse oximetry were not conducted because of the narrow range of values. The *t* tests were performed to compare differences between subgroups using Microsoft Excel. *P* values are given for subgroup analyses, with a threshold of .05 considered statistically significant.

## Results

### Demographics

A total of 19 patients were enrolled in the study. The average age was 79.6 years, and the patient group was almost equally divided by gender (10 female, 9 male). Patients’ FEV_1_% (forced expiratory volume in 1 second as % of predicted for the patient) at study entry averaged 56.2% of predicted (range 23%-113%), and FEV_1_/forced vital capacity averaged 65%. The majority of patients (n=10, 53%) fell into the severe disease category, as indicated by the Global Initiative for Chronic Obstructive Lung Disease (GOLD) criteria [9]. A total of 6 patients (32%) were in the GOLD 2, or moderate category, and 2 (11%) were in the mild category; 1 patient (5%) was categorized as GOLD 4, or very severe. This distribution is not surprising as patients were required to be using nebulizers, which are not typically prescribed for mild disease. A total of 5 patients had a caregiver assist them with many of the activities, including equipment use and symptom/medication input (Table 1).

**Table 1:** Demographics and baseline characteristics of the study population.

Demographic characteristic		Value (N=19)
Age in years, mean (range)		79.6 (65-95)
**Gender, n (%)**		
	Male	9 (47)
	Female	10 (53)
**GOLD^a^ category, n (%)^b^**		
	GOLD 1: mild (FEV_1_^c^ ≥80% predicted)	2 (11)
	GOLD 2: moderate (FEV_1_ ≥50-79%)	6 (32)
	GOLD 3: severe (FEV_1_ ≥30-49%)	10 (53)
	GOLD 4: very severe (FEV_1_ <30%)	1 (5)
	FEV_1_%^d^, mean (range)	56.2 (23-113)
	FEV_1_/forced vital capacity, mean	65.3
	Home oxygen use, n (%)	9 (47)
	Pack years, mean (range)	51.6 (10-100)
**Independent versus assisted use of Wellinks mHealth solution, n (%)**		
	Independent use	14 (74)
	Assisted use (with help from a caregiver or home health aide)	5 (26)

^a^GOLD: Global Initiative for Chronic Obstructive Lung Disease.

^b^All patients with FEV_1_/forced vital capacity <0.70.

^c^FEV_1_: forced expiratory volume in 1 second.

^d^FEV_1_%: forced expiratory volume in 1 second as % of predicted for the patient.

### Patient Use of the Intervention

Participants used the pulse oximeter 4.2 times/week and the spirometer 2.5 times per week on average (Table 2). All 19 patients achieved the weekly goal of at least one pulse oximetry reading per week over 8 weeks, and 15 patients met or exceeded the goal of 1 spirometry reading per week. On average, patients’ medication use was entered into the app 9 times/week, and symptoms entered 1.2 times/week.

Differences in participation did not significantly differ by COPD severity, age, or gender (Table 3).

**Figure 5:**
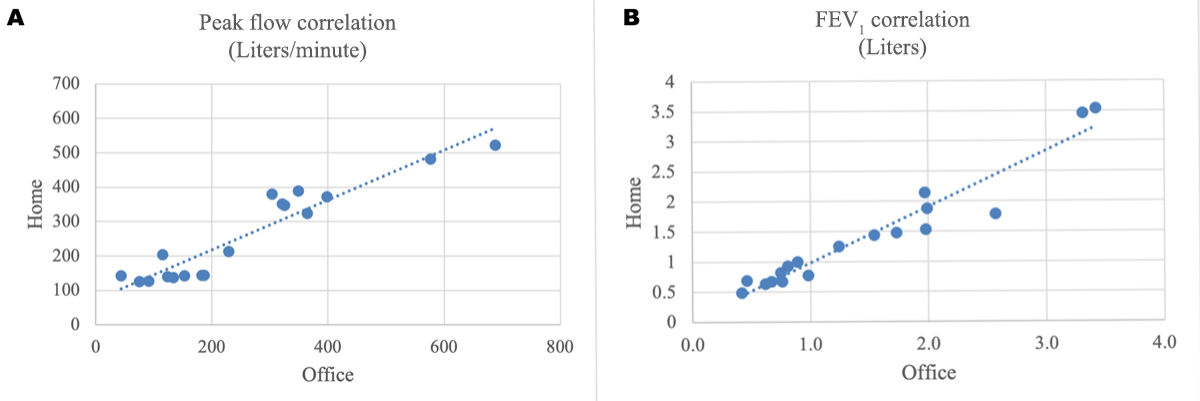
The correlation of home versus office assessments of (A) peak flow and (B) FEV_1_. FEV_1_: forced expiratory volume in 1 second.

There was a strong correlation between the FEV_1_ (r=0.96) and peak flow (r=0.94) measurements recorded by the spirometer compared with the measurements recorded by the physician during the office visit closest in time to the at-home collected information (Figure 5).

**Table 2:** Study participant interaction with the mobile health platform.

Data collected	Mean number of app entries/week (range of number of app entries/week)
FEV_1_^a^ by spirometer	2.5 (1-7)
Blood oxygenation by pulse oximeter	4.2 (1-12)
Medication use	9.0 (1-25.1)
Nebulizer use	1.9 (0-11.9)
Symptoms	1.2 (0-5.6)

^a^FEV_1_: forced expiratory volume in 1 second.

**Table 3:** Participation by gender, age, and COPD^a^ severity.

Demographic characteristic		n	Spirometry^b^	Pulse oximetry^b^	Medications^c^	Symptoms^c^
**Gender**						
	Male	9	19.3	35.2	10.3	6.5
	Female	10	21.1	32.6	21.1	12.2
	*P* value		.81	.84	.37	.22
**Age**						
	<80	11	25.4	41.6	22.8	11.6
	≥80	8	13.0	23.5	5.3	5.8
	*P* value		.09	.15	.14	.22
**COPD severity**						
	Mild + moderate	8	19.8	28.5	17.1	5.5
	Severe + very severe	11	20.5	37.9	14.1	11.9
	*P* value		.93	.46	.81	.17

^a^COPD: chronic obstructive pulmonary disease.

^b^Data are presented as mean number of uses/week.

^c^Data are presented as mean number of times reported.

### Patient Engagement and Satisfaction

**Figure 6:**
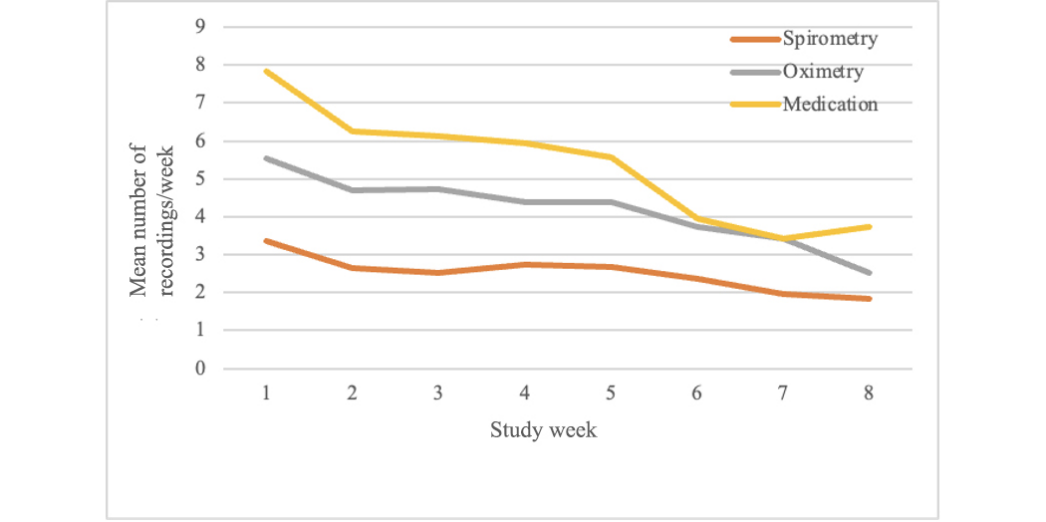
Engagement, measured in app uses or recordings/week, fell over the course of the 8-week study, but remained above the baseline requested of 1 use/week for spirometry and oximetry recordings.

The average number of recordings for spirometry, oximetry, and medication usage declined over the 8-week interval by varying degrees (Figure 6). Medication use entries fell from 7.8 times/week to 3.7 times/week (a reduction of 52.3%), oximetry recordings from 5.5 times/week to 2.5 times/week (a reduction of 54.2%), and spirometry recordings from 3.4 times/week to 1.8 times/week (a reduction of 45.4%).

The satisfaction survey administered is presented as Multimedia Appendix 1. A total of 16 patients were available to take this survey. Most patients (15/16, 94%) either agreed or strongly agreed that the Wellinks app was easy to use and valuable (13/16, 81%). Most (15/16, 94%) found that it was valuable to be able to take spirometry and pulse oximetry measurements at home. The symptom logging function was found to be moderately valuable (11/16, 69%) as was the medication schedule (10/16, 63%). Patients expressed interest (12/16, 75%) in adding a physician or care team messaging component to the app. In general, the patients did not feel that the app enhanced their knowledge of COPD or the connection with their doctor. The NPS generated by the patients in this study was 59.

### Adverse Events

There were no issues reported by either patients or the principal investigator related to the safety of the patients or the performance of the devices.

## Discussion

### Principal Findings

COPD is a chronic condition where the potential for rapid deterioration due to exacerbations is an ever-present danger [6,7,9]. For this reason, it can be a very difficult condition to manage as adjustments in care are dependent on the patient recognizing and reporting changes from baseline. While various devices exist to enable monitoring of specific individual parameters, an app that allows patients and physicians to track multiple modalities simultaneously, and remotely, would result in the most complete clinical picture available from the patient’s daily life.

In this pilot study, we observed that study participants were both willing to use the Wellinks mHealth solution and able to do so effectively, performing both spirometry and oximetry measurements far more often than requested, and logging medication use and symptoms frequently without being prompted. Patients engaged with the Wellinks platform regardless of disease severity, gender, or age. Notably, the correlations of peak flow and FEV_1_ measurements taken by the patient at home and those performed in the office were very high. This has been a technical failure in prior studies, where use of the spirometer in an outpatient setting and without the assistance of a health care practitioner has resulted in inaccurate results [20,21].

Age may be a concern in the adoption of an mHealth system in patients with COPD, as COPD is largely a disease of the elderly who may have a lower digital literacy level, less access to smart devices, and overall, a lower comfort level with technology. A 2018 study of 638,330 Medicare beneficiaries found that 40.9% lacked a smartphone with a wireless data plan, and 26.3% lacked any type of digital access (either a smartphone or a home desktop or laptop computer with a high-speed internet connection) [22].

However, the COVID-19 pandemic has changed the way the elderly view technology. In a recent study conducted by the American Association for Retired People (AARP), 45% of individuals over 60 years viewed technology far more positively than they did prior to COVID. Among those 70 and over, 73% reported having high-speed internet access at home. Ownership of smart technology, including smartphones, continues to increase among those over age 70, and accessing health care services and health information is among the top 10 activities conducted on their smartphones. This represents a significant increase over 2019 [15].

When surveyed in this study, patients found the Wellinks platform to be both easy to use and valuable. These 2 factors have been found to be among the highest predictors of intent to use a medical app within a senior population, as found in a recent study of 364 older adults with an average age of 75 years [23]. In our study, the 2 factors with the lowest scores were the ability to learn about COPD via the app, and enhanced connection to the physician. While access to educational content and 2-way communication are planned for the next-generation Wellinks platform, the study version of the app did not provide any educational material, nor was there any provision for direct communication between the patient and the physician. Patients or their caregivers had the opportunity to give the interviewer feedback about the Wellinks Platform at the end of the study period. Some patients reported technical difficulties, or the need for more clear instructions; others suggested ideas for improvement, such as integration of educational content to teach patients about the device readings and what the numbers meant. Several patients expressed an appreciation for the ability to track spirometry and oximetry values in real time.

NPS has proven to be a remarkably powerful assessment of customer satisfaction across multiple industries [24] and is now increasingly used in the health care setting due to its ease of administration and higher response rates. NPS surveys can be readied more quickly than email or phone surveys and are less time-consuming for patients, and thus more likely to garner a response [25]. NPS methodology is based on research that cross-references survey responses with actual consumer behavior, demonstrating that a strong personal recommendation of a company or a service to one’s family and friends was a key measure of satisfaction [26], and directly correlated with uptake in the market [19]. An NPS of 59, as seen with the Wellinks platform, indicates a very high level of patient satisfaction [19].

### Limitations

First, it must be noted that mobile and eHealth innovations are often subject to a high rate of attrition, for various reasons; for example, the novelty wears off over time, or patients have difficulty fitting the technology into their everyday lives. This can make it difficult to assess these types of technologies [27,28]. In the case of the Wellinks platform, the base requirement for spirometry and oximetry was only 1 use/week. Therefore, we believe the decline in usage for these 2 parameters is more reflective of hyperutilization toward the beginning of the study which then reverted to the anticipated baseline. The reduction in use of the medications feature may be reflective of the high medication burden for patients with COPD in general. Yet, even the reduced usage rate represents more information than would normally be available to clinicians.

Second, this study was a small pilot with all patients selected by 1 physician at 1 pulmonology practice. Certain study parameters limited our study population, for example, the requirements of access to an iPhone and of patient nebulizer use. The fact that the study recruited from 1 small geographic area limits its generalizability as it may not reflect a diverse population.

Third, patient engagement may be artificially higher than expected in a real-world situation due to the Hawthorne effect (where individuals modify their behavior because they are being observed) [29]. In this study, patients were recruited by the principal investigator and were aware that their use of apps and monitoring devices was being recorded. This may have motivated the patients to use the app more consistently than if they were not aware that they were being observed. The slow decline in usage over 8 weeks may reflect that effect over time.

Lastly, while useful data regarding patient health status were provided via the patient report, there was no formal requirement for ongoing physician assessment of the data provided. The study was not designed to show improvement in COPD, only that patients would utilize the Wellinks system. Consequently, there were no interventions taken, and the study was not powered to show improvement in clinical outcomes or pharmacoeconomic impact.

### Future Directions

This important pilot study proves that the multimodal nature of the Wellinks Platform is feasible and that appropriate utilization and engagement by the target population is possible. The information collected will direct the continued development of the Wellinks Platform, including features such as virtual pulmonary rehabilitation and health coaching, and has the potential to create value for patients and the health care system. Important questions remain: can this platform improve the quality of care for patients with COPD? Can it improve clinical outcomes such as a reduction of hospital readmissions and cost and improving patient quality of life? These questions will be addressed in a planned future clinical study.

### Conclusions

The Wellinks platform is a novel platform designed for patients with COPD that consolidates multimodal biometric data and patient entries into a single, streamlined source of data including trends over time and is intended for use by both patients and providers. Patients were eager to engage with the platform and found it to be useful and valuable. Patient engagement was high across disease severity, age, and gender groupings. Given the difficulty of managing this complex, chronic disease, the Wellinks platform has the potential to improve patient engagement and outcomes, ultimately reducing COPD-related events and expenses.

## Appendix

Multimedia Appendix 1 Responses to the Patient Satisfaction questionnaire (n=16). Patients submitted an answer on a 5-point scale ranging from strongly disagree to strongly agree.

## Abbreviations

AARP: American Association for Retired People
COPD: chronic obstructive pulmonary disease
FEV_1_: forced expiratory volume in 1 second
FEV_1_%: forced expiratory volume in 1 second as % of predicted for the patient
GOLD: Global Initiative for Chronic Obstructive Lung Disease
HIPAA: Health Insurance Portability and Accountability Act
IRB: Institutional Review Board
NPS: Net Promoter Score
PRN: pro re nata (as needed)
SpO_2_: peripheral oxygen saturation
