# Supplementary material for: An Integrated, Multimodal, Digital Health Solution for Chronic Obstructive Pulmonary Disease: Prospective Observational Pilot Study
Source: JMIR Form Res. 2022 Mar 17;6(3):e34758. doi: 10.2196/34758 (PMC8972120; doi:10.2196/34758)
Supplement: Multimedia Appendix 1 [file formative_v6i3e34758_app1.docx]

| Question | Strongly Disagree  n (%) | Disagree  n (%) | Neither Agree nor Disagree  n (%) | Agree  n (%) | Strongly Agree  n (%) |
| --- | --- | --- | --- | --- | --- |
| I found the Wellinks app easy to use. | 0 (0) | 1 (6) | 0 (0) | 10 (62) | 5 (31) |
| Overall, I find the Wellinks app to be valuable. | 0 (0) | 1 (6) | 2 (13) | 9 (56) | 4 (25) |
| I think being able to take and log spirometry measurements at home is valuable. | 0 (0) | 0 (0) | 1 (6) | 9 (56) | 6 (38) |
| I think being able to take and log pulse oximeter measurements at home is valuable. | 0 (0) | 1 (6) | 0 (0) | 7 (44) | 8 (50) |
| I think being able to track and log my symptoms in the app is valuable. | 0 (0) | 2 (13) | 3 (19) | 5 (31) | 6 (38) |
| I think having my medication schedule in the app is valuable. | 0 (0) | 3 (19) | 3 (19) | 5 (31) | 5 (31) |
| I would like to be able to message my doctor or caregiver through the app. | 0 (0) | 3 (19) | 1 (6) | 7 (44) | 5 (31) |
| Using the Wellinks app has helped me to learn more about my COPD. | 0 (0) | 5 (31) | 5 (31) | 3 (19) | 3 (19) |
| Using the Wellinks app has strengthened my connection to my doctor. | 0 (0) | 7 (44) | 6 (38) | 2 (13) | 1 (6) |
